# Metabolic heterogeneity at the onset of type 2 diabetes: phenotypes of insulin resistance and β-cell function in the Kazakh population

**DOI:** 10.3389/fendo.2026.1824856

**Published:** 2026-08-25

**Authors:** Bibigul Tleumagambetova, Raikul Kosmuratova, Khatimya Kudabayeva, Yerlan Bazargaliyev

**Affiliations:** Department of Internal Diseases No 1, West Kazakhstan Marat Ospanov Medical University, Aktobe, Kazakhstan

**Keywords:** cluster analysis, HOMA2 model, insulin resistance, Kazakhstan, metabolic phenotypes, newly diagnosed diabetes, type 2 diabetes mellitus, β-cell dysfunction

## Abstract

**Background:**

Type 2 diabetes mellitus (T2DM) is increasingly recognized as a heterogeneous metabolic disorder characterized by varying contributions of insulin resistance and pancreatic β-cell dysfunction. However, data on early metabolic phenotypes at diagnosis remain limited in Central Asian populations.

**Methods:**

In this cross-sectional study, 240 adults with newly diagnosed T2DM were recruited from primary health care facilities in the Aktobe region of Kazakhstan between May 2024 and January 2025. Islet autoantibodies (GAD, IA-2, ZnT8, and ICA) were measured to exclude autoimmune diabetes. After exclusion of seropositive individuals, 178 autoantibody-negative participants were analyzed. Insulin resistance and β-cell function were assessed using the HOMA2 model, and metabolic phenotypes were explored using unsupervised clustering based on BMI, HbA1c, HOMA2-IR, HOMA2-%B, and basal C-peptide.

**Results:**

Among the 178 autoantibody-negative individuals, insulin resistance defined as HOMA2-IR ≥1.6 was observed in 59% of participants, whereas β-cell dysfunction defined as basal C-peptide <1.70 ng/mL was present in 30.9%. Unsupervised clustering identified four distinct metabolic phenotypes. Cluster 1 represented the largest subgroup (42.1%) and demonstrated a relatively balanced metabolic profile. Cluster 2 (12.9%) was characterized by obesity-associated hyperinsulinemia with elevated BMI and C-peptide levels. Cluster 3 (25.3%) showed marked hyperglycemia accompanied by reduced β-cell function, whereas Cluster 4 (19.7%) combined increased BMI with relatively preserved β-cell function and lower glycemic burden.

**Conclusions:**

Newly diagnosed T2DM in the Kazakh population demonstrates substantial metabolic heterogeneity already at the time of diagnosis. Identification of distinct metabolic phenotypes using routinely available clinical variables may provide a framework for future studies evaluating precision-based approaches to diabetes management.

## 1 Introduction

According to the 11th edition of the International Diabetes Federation (IDF) Diabetes Atlas, the global prevalence of diabetes mellitus reached approximately 589 million adults aged 20–79 years in 2024 (11.1% of the adult population) and is projected to exceed 850 million by 2050, underscoring the escalating global burden of type 2 diabetes mellitus (T2DM) (1). In Kazakhstan, the burden of type 2 diabetes mellitus continues to grow, with recent national data indicating a rising prevalence accompanied by substantial systemic gaps in early detection and management, underscoring the need for population-based characterization of newly diagnosed cases (2).

Given the high proportion of newly diagnosed T2DM, early metabolic characterization at the time of diagnosis is of particular importance. Evaluation of key components of glucose homeostasis at this stage may provide insight into underlying pathophysiological heterogeneity, particularly with respect to insulin resistance and pancreatic β-cell function, before substantial disease progression or treatment-related effects occur (3). Such early characterization is essential for improving the understanding of disease mechanisms across different age groups, especially in populations with a high burden of newly detected diabetes (4). This variability reflects differing contributions of insulin resistance and pancreatic β-cell dysfunction across individuals.

In this context, T2DM is increasingly recognized as a heterogeneous metabolic disorder rather than a single uniform disease entity (5). At the time of diagnosis, this heterogeneity can be pragmatically explored through the assessment of insulin sensitivity and β-cell secretory capacity using validated surrogate indices (6). Although gold-standard methods such as the hyperinsulinemic-euglycemic clamp or intravenous glucose tolerance testing provide precise physiological measurements, their complexity and cost limit their applicability in large-scale or routine clinical settings (7). In contrast, surrogate indices derived from fasting glucose and insulin concentrations offer a feasible and widely accepted approach for characterizing metabolic phenotypes in epidemiological and clinical research.

Among these approaches, the original Homeostasis Model Assessment proposed by Matthews et al. was based on a simplified mathematical approximation of glucose-insulin interactions (8). In contrast, the updated HOMA2 model is a computer-based approach that incorporates non-linear physiological relationships, allowing for a more accurate representation of insulin-glucose homeostasis than the original HOMA1 formulation (9). By accounting for variations in hepatic and peripheral insulin resistance, HOMA2 provides refined estimates of pancreatic β-cell function, insulin resistance, and insulin sensitivity, expressed as HOMA2-%B, HOMA2-IR, and HOMA2-%S, respectively, with insulin sensitivity defined as the reciprocal of insulin resistance (10). This integrated assessment enables a comprehensive characterization of metabolic phenotypes at the time of diagnosis and is particularly suitable for evaluating early pathophysiological heterogeneity in individuals with dysglycemia.

However, data on early metabolic phenotypes based on insulin resistance and β-cell function at the time of diagnosis remain limited in Central Asian populations, including Kazakhstan, where a substantial proportion of T2DM cases are newly detected. Therefore, the aim of this cross-sectional study was to evaluate insulin resistance and pancreatic β-cell function at the time of diagnosis using fasting-based surrogate indices (HOMA2-%B, HOMA2-%S, and HOMA2-IR) and to characterize early metabolic phenotypes in adults with newly diagnosed T2DM in the Kazakh population.

## 2 Materials and methods

### 2.1 Study design, setting, and study population

This descriptive cross-sectional study was conducted to characterize insulin resistance and pancreatic β-cell functional impairment in adults with newly diagnosed T2DM. Participants were recruited from primary health care facilities in the Aktobe region, Republic of Kazakhstan, between May 1, 2024, and January 4, 2025. Inclusion criteria were: age 25–75 years at diagnosis; newly diagnosed T2DM according to ADA criteria (HbA1c ≥6.5% and/or plasma glucose ≥11.1 mmol/L); absence of insulin therapy within the first 6 months after diagnosis; and no history of diabetic ketoacidosis. Exclusion criteria were: type 1 diabetes mellitus or secondary diabetes; acute or chronic infections potentially affecting glycemic status within 4 weeks prior to enrollment; history of malignancy; pregnancy or breastfeeding; and severe comorbid conditions limiting participation. The diagnosis of T2DM and eligibility were independently confirmed by two endocrinologists prior to inclusion.

### 2.2 Sample size

A total of 240 patients were assessed for eligibility. Sample size estimation was performed using Epi Info™ software (version 7.2.5.0; CDC, USA). For analyses focusing on metabolic phenotypes of non-autoimmune T2DM, individuals positive for at least one islet autoantibody, including antibodies to glutamic acid decarboxylase (GAD), tyrosine phosphatase (IA-2), zinc transporter 8 (ZnT8), or islet cell antibodies (ICA), were excluded. The final analytic cohort comprised 178 islet autoantibody-negative participants.

### 2.3 Anthropometric assessment

Anthropometric measurements were performed following standardized international protocols (11). Body weight was measured twice using calibrated digital medical scales, with participants wearing light clothing and no shoes; values were recorded to the nearest 0.1 kg. Height was measured using a wall-mounted stadiometer to the nearest 0.1 cm, with participants standing upright in the Frankfurt horizontal plane. BMI was measured according to standardized procedures and expressed in kg/m². Interpretation was based on WHO (12).

### 2.4 Laboratory measurements

Venous blood samples were collected after an overnight fast of 8–12 hours using vacuum collection systems. Samples were centrifuged and serum aliquots were stored at −20 °C until analysis. Fasting plasma glucose was measured using the glucose oxidase method on an automated biochemistry analyzer. HbA1c was determined from whole blood using a standardized latex agglutination method. Serum insulin and C-peptide concentrations were measured using chemiluminescent immunoassays. To exclude autoimmune diabetes, serum islet autoantibodies (GAD, IA-2, ZnT8, and ICA) were assessed. All autoantibodies were measured using enzyme-linked immunosorbent assay (ELISA) kits according to the manufacturers’ instructions.

### 2.5 Assessment of insulin resistance and β-cell function

Insulin resistance and β-cell function were evaluated using the HOMA2 model, expressed as: HOMA2-IR - insulin resistance, HOMA2-%B - β-cell function, and HOMA2-%S - insulin sensitivity. Calculations were performed using the HOMA2 Calculator (version 2.2.4) developed by the Diabetes Trials Unit, University of Oxford (available at: https://www.dtu.ox.ac.uk/homacalculator/). HOMA2-IR, HOMA2-%B and HOMA2-%S were calculated from fasting plasma glucose and fasting serum insulin concentrations.

### 2.6 Definition of metabolic phenotypes and cluster analysis

Metabolic phenotypes were defined using clinically interpretable thresholds commonly used in metabolic studies. Insulin resistance was defined as HOMA2-IR ≥1.6, and β-cell dysfunction as basal C-peptide <1.70 ng/mL. To further explore metabolic heterogeneity, unsupervised clustering was performed using the following variables: BMI, HbA1c, HOMA2-%B, HOMA2-IR, and basal C-peptide.

### 2.7 Statistical analysis

Continuous variables were assessed for distributional characteristics and are presented as median and interquartile range [IQR]. Categorical variables are presented as frequencies and percentages. Differences in continuous variables across clusters were assessed using the Kruskal–Wallis test, whereas categorical variables were compared using Pearson’s χ² test. All tests were two-sided, and p < 0.05 was considered statistically significant.

Clustering variables were standardized to z-scores before analysis. Hierarchical clustering was performed using Ward’s minimum variance method and squared Euclidean distance. The number of clusters was evaluated using dendrogram inspection, changes in agglomeration coefficients, and average silhouette width. The four-cluster solution was subsequently examined using K-means clustering.

Internal cluster stability was assessed using non-parametric bootstrap resampling with 500 replicates. In each replicate, clustering was repeated, and cluster-wise similarity with the original partition was assessed using the Jaccard coefficient. Mean Jaccard coefficients ≥0.75 were interpreted as indicating good stability, whereas values of 0.60–0.75 indicated moderate stability.

Statistical analyses were performed using IBM SPSS Statistics version 26.0 (IBM Corp., Armonk, NY, USA). Data visualization and cluster validation were performed using R software (version 4.4.x) in RStudio (version 2026.01.1 + 403).

### 2.8 Ethical approval

The study was conducted in accordance with the Declaration of Helsinki. Written informed consent was obtained from all participants prior to enrollment. The protocol was approved by the Institutional Bioethics Committee of West Kazakhstan Marat Ospanov Medical University (Protocol № 10, approval number 10.16.01, dated 31 October 2023).

## 3 Results

A total of 240 patients with recent-onset T2DM were examined. Among them, 178 patients (74.2%) were negative for all tested islet autoantibodies, whereas 62 patients (25.8%) were seropositive for at least one autoantibody. After exclusion of islet autoantibody-positive individuals, the final analytic cohort comprised 178 patients who were included in the subsequent analyses of metabolic phenotypes. The baseline clinical and metabolic characteristics of these islet autoantibody-negative patients are summarized in **Table 1**.

**Table 1:** Baseline clinical and metabolic characteristics of islet autoantibody-negative patients with newly diagnosed type 2 diabetes mellitus.

Variable	Median [IQR]
Age, years	51.0 [41.0-60.0]
BMI, kg/m²	29.4 [25.9-33.7]
HbA1c, %	7.76 [6.50-10.79]
Basal C-peptide, ng/mL	2.32 [1.49-3.38]
HOMA2-%B	52.8 [29.5-110.9]
HOMA2-%S	54.4 [33.0-81.4]
HOMA2-IR	1.86 [1.24-3.05]

Among the 178 islet autoantibody-negative patients, HOMA2-IR ≥1.6 was present in 105 individuals (59%), while 73 patients (41%) had HOMA2-IR values <1.6. Basal C-peptide concentrations <1.70 ng/mL, used as an exploratory indicator of reduced β-cell secretory capacity, were observed in 55 patients (30.9%), whereas 123 patients (69.1%) had concentrations ≥1.70 ng/mL. The clinical and metabolic characteristics of these threshold-defined subgroups are presented in **Supplementary Table 1**.

Although the above threshold-based stratification revealed clinically meaningful patterns of β-cell impairment, it relied on predefined cut-off values and therefore could not determine whether these categories represented natural biological groupings or artificial divisions along a continuum. To address this limitation and to evaluate whether patients spontaneously segregate into metabolically distinct subgroups, we next applied an unsupervised clustering approach using standardized metabolic variables incorporating both insulin resistance and β-cell function.

**Figure 1:**
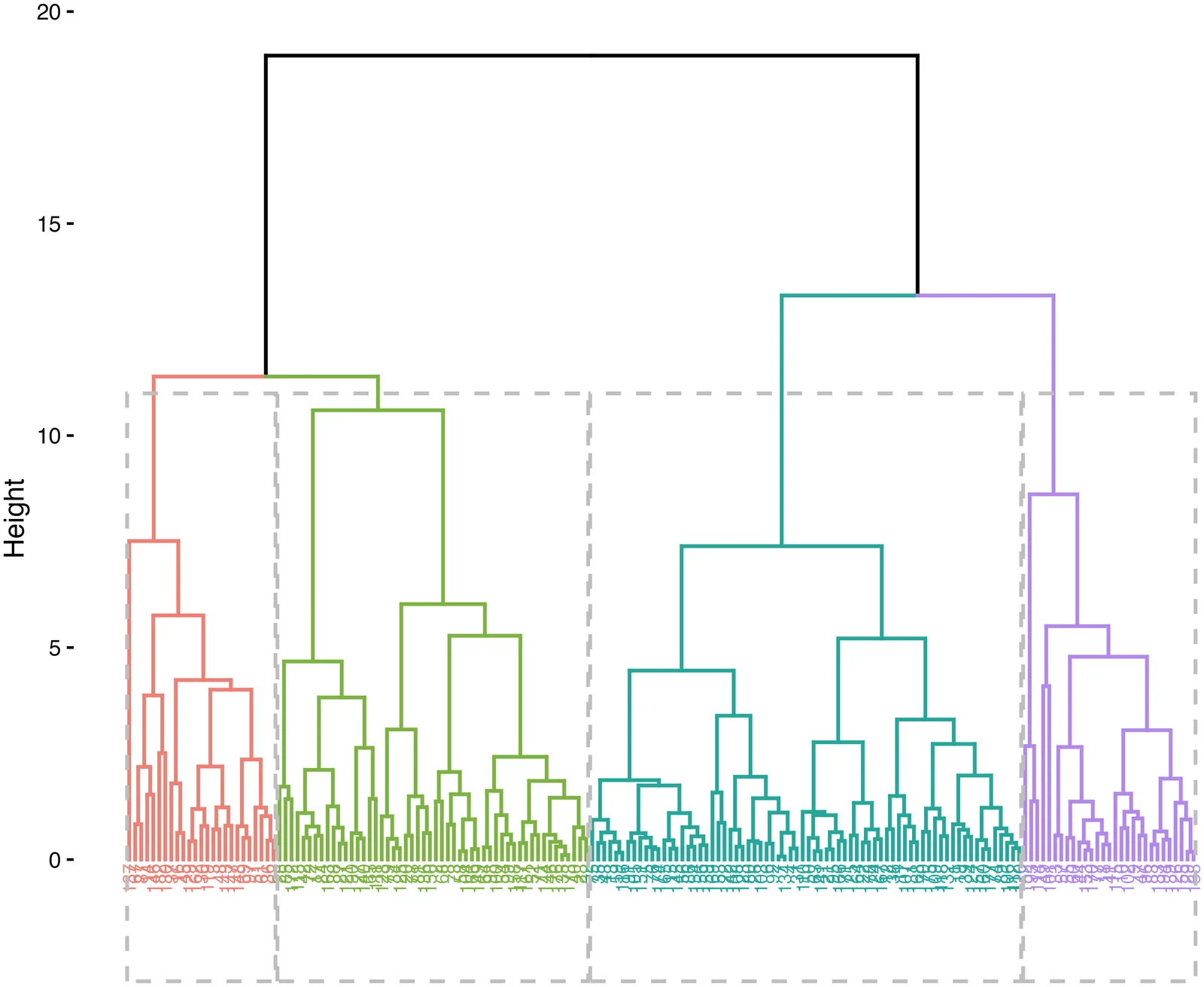
Hierarchical clustering dendrogram of metabolic variables suggesting a four-cluster solution in newly diagnosed T2DM.

Hierarchical clustering using Ward’s method with squared Euclidean distance was performed on standardized metabolic variables (HOMA2-%B, HOMA2-IR, basal C-peptide, BMI, and HbA1c). All variables were standardized (z-scores) prior to clustering to ensure comparability across scales. Inspection of the agglomeration schedule revealed a gradual increase in fusion coefficients followed by a marked jump at later stages, suggesting the presence of a stable three- to four-group structure. Accordingly, a four-group solution was selected and further evaluated using K-means clustering (**Figure 1**).

**Figure 2:**
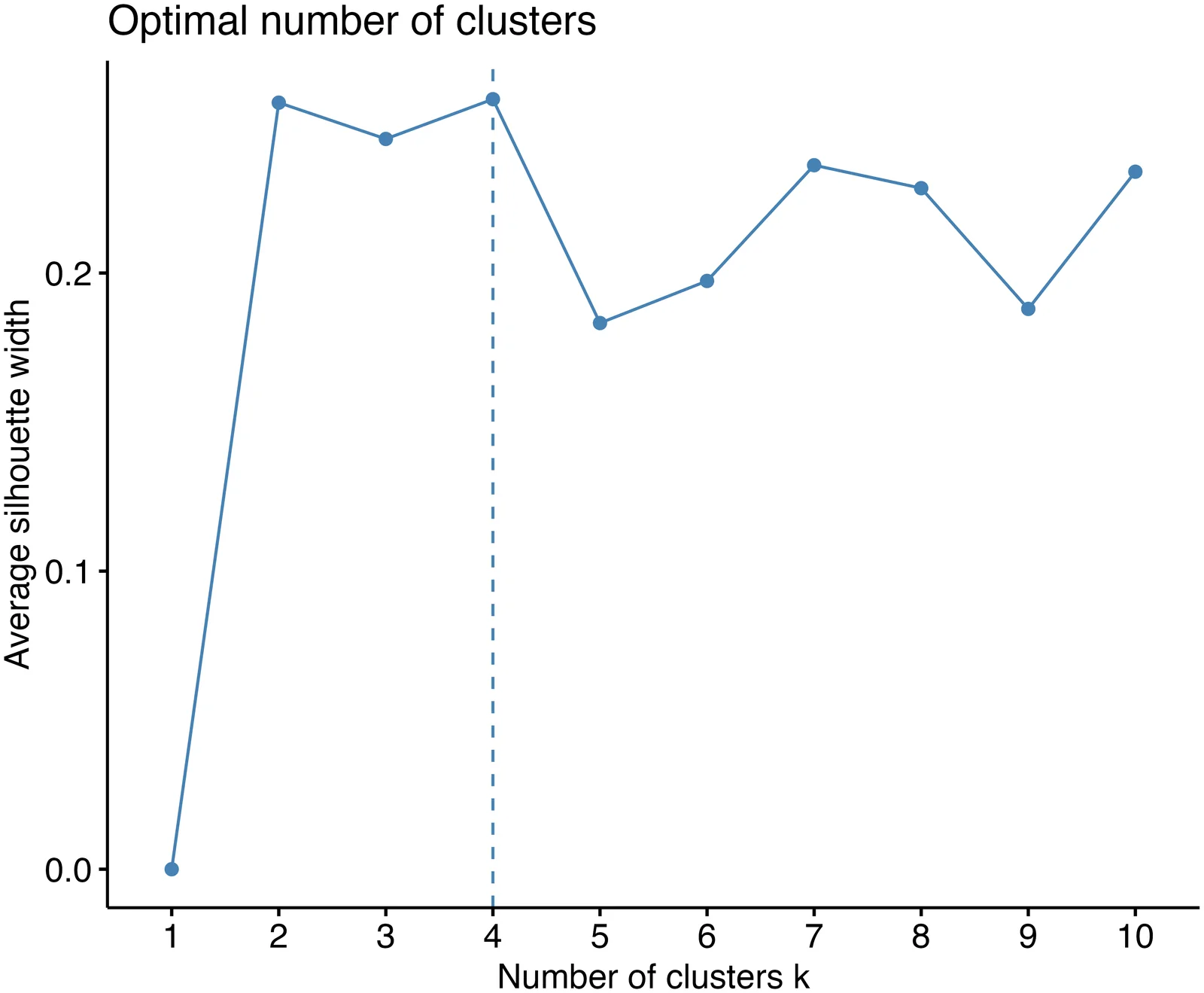
Determination of the optimal number of clusters using silhouette analysis.

Silhouette analysis demonstrated the highest average silhouette width at k = 4, confirming the optimal group structure identified by hierarchical clustering(**Figure 2**). The highest average silhouette width was observed at k = 4 (0.31). Although the absolute silhouette value indicated only modest separation between clusters, it was higher than that obtained for the alternative solutions and therefore supported selection of the four-cluster model for further exploratory analysis.

**Figure 3:**
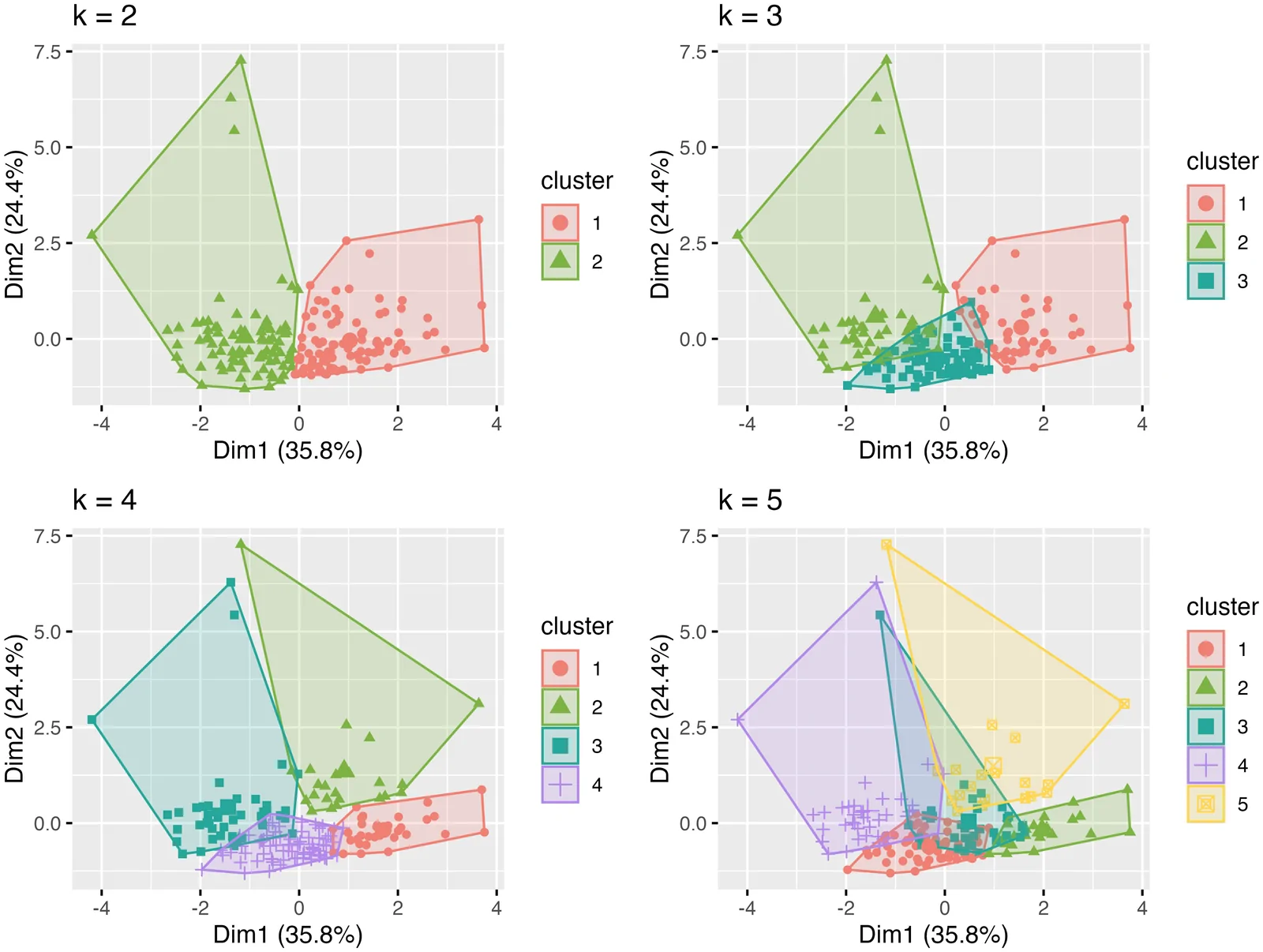
Validation of phenotype structure using K-means clustering. Projection of individuals onto the first two principal components for alternative clustering solutions (k = 2-5).

To assess the robustness and separability of the derived groups, the clustering results were visualized in principal component space. Projection onto the first two principal components demonstrated clear spatial separation between groups with minimal overlap compared with alternative solutions (k = 2, 3, and 5), supporting the stability and interpretability of the four-phenotype model (**Figure 3**). The final K-means solution was therefore retained and interpreted as distinct metabolic phenotypes.

To evaluate the robustness of the identified metabolic clusters, internal validation was performed using a non-parametric bootstrap resampling procedure. For each bootstrap sample, the clustering algorithm was re-applied and the resulting partitions were compared against the original solution using the Jaccard similarity coefficient. Cluster-wise stability was quantified as the mean Jaccard index across all replicates, with values ≥ 0.75 considered indicative of a stable and reproducible cluster structure, in line with previously established recommendations.

**Figure 4:**
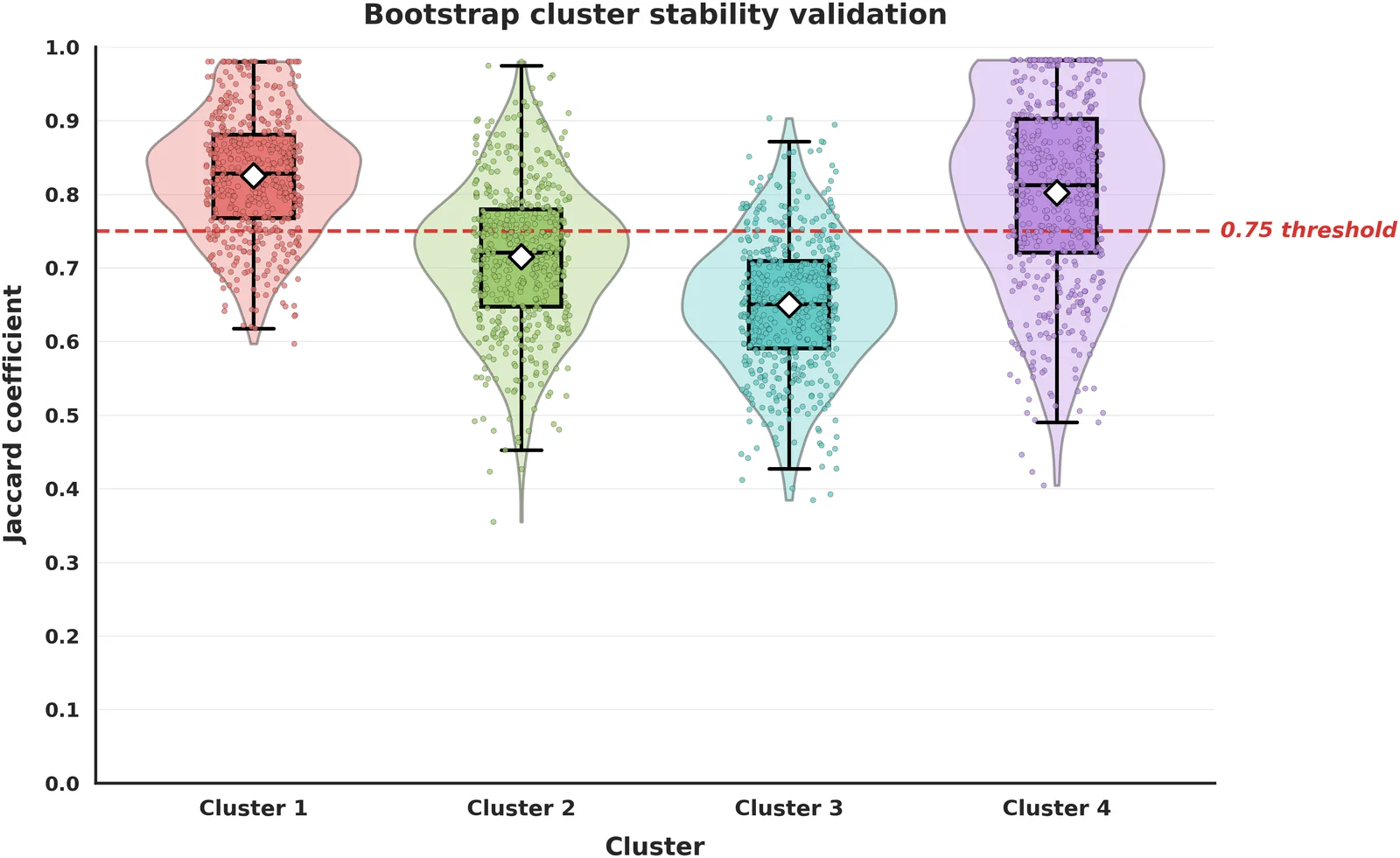
Bootstrap-based internal validation of the four-cluster solution. Violin plots depict the distribution of Jaccard similarity coefficients across 500 bootstrap replicates for each cluster.

The mean Jaccard coefficients were 0.825 for Cluster 1, 0.715 for Cluster 2, 0.650 for Cluster 3, and 0.802 for Cluster 4 (**Figure 4**). Clusters 1 and 4 exceeded the predefined 0.75 stability threshold, indicating a highly reproducible assignment of individuals across resampled datasets and supporting these subgroups as robust metabolic phenotypes. Clusters 2 and 3, although falling below the strict cut-off, still demonstrated moderate stability (mean Jaccard > 0.60), suggesting that their core structure is preserved but that a proportion of borderline cases may be reassigned between adjacent phenotypes - most plausibly reflecting the continuum between insulin-resistant and β-cell-deficient states at diagnosis.

Overall, the bootstrap analysis demonstrated good stability for Clusters 1 and 4 and moderate stability for Clusters 2 and 3. These findings provide partial support for the reproducibility of the four-cluster solution while indicating that the boundaries between some metabolic profiles remain uncertain.

The study cohort was distributed across four distinct metabolic clusters, with Cluster 1 representing the largest subgroup and Cluster 2 the smallest, while Clusters 3 and 4 demonstrated intermediate representation (**Table 2**).

**Table 2:** Distribution of metabolic clusters in the study cohort.

Cluster	n	Percentage of total cohort (%)
Cluster 1	75	42.1%
Cluster 2	23	12.9%
Cluster 3	45	25.3%
Cluster 4	35	19.7%

**Figure 5:**
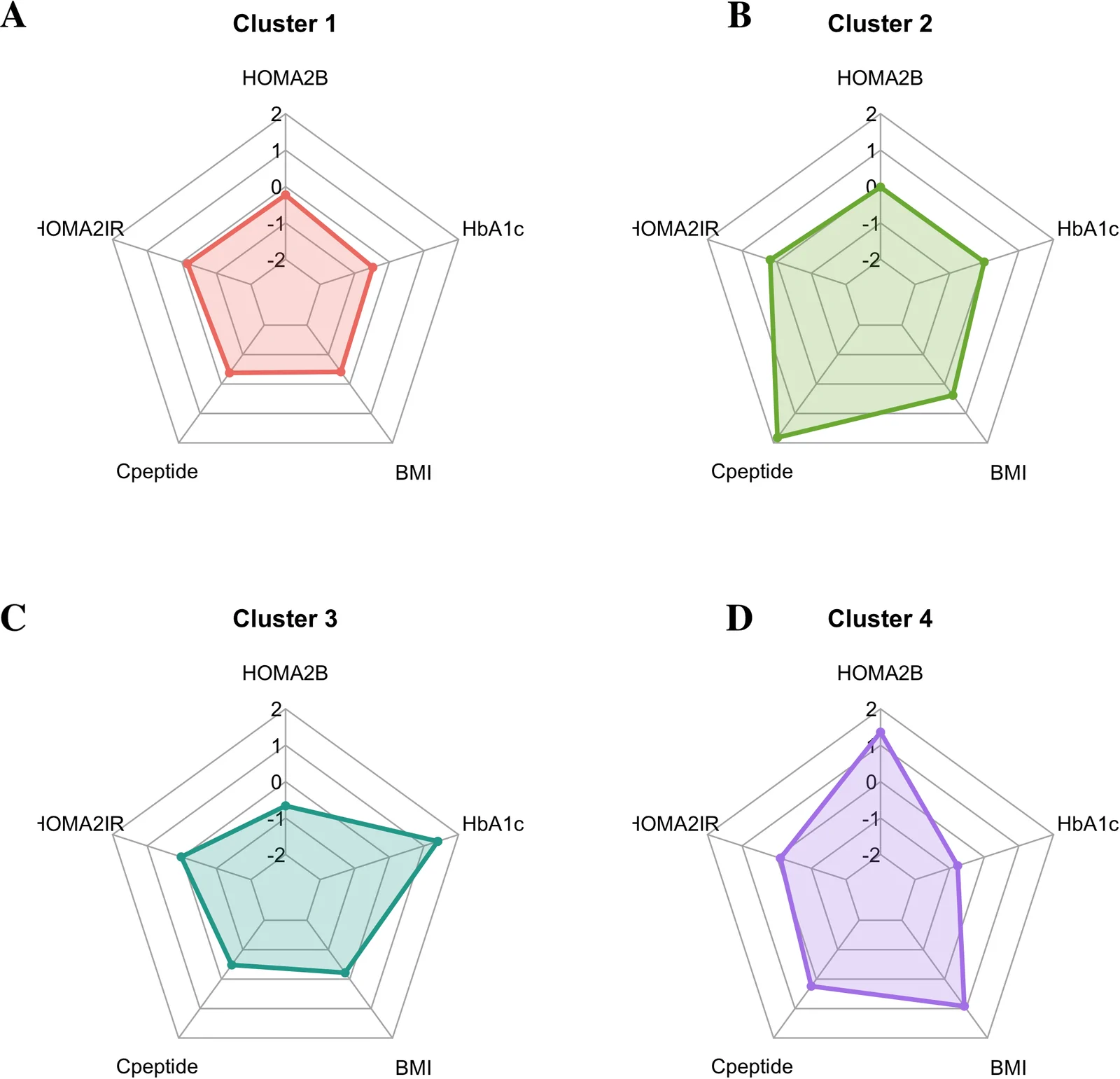
Radar plot representation of metabolic cluster profiles based on standardized HOMA2-%B, HOMA2-IR, C-peptide, BMI, and HbA1c values. Panels **(A–D)** correspond to clusters 1–4, respectively.

Four distinct metabolic patterns were identified across the analyzed variables. Cluster 1 represented a comparatively moderate metabolic profile, with lower HOMA2-IR, intermediate glycemic burden, and no single dominant metabolic abnormality. (**Figure 5A**). Cluster 2 expanded primarily along the C-peptide and BMI dimensions, while HOMA2-%B, HOMA2-IR, and HbA1c remained centered within the overall distribution (**Figure 5B**). Cluster 3 was driven by the HbA1c axis, forming a profile dominated by glycemic exposure, whereas C-peptide and HOMA2-%B appeared attenuated and BMI and HOMA2-IR occupied intermediate positions (**Figure 5C**). Cluster 4 showed a prominent projection along the HOMA2-%B and BMI axes, accompanied by comparatively subdued HbA1c and HOMA2-IR and a moderate C-peptide position (**Figure 5D**).

Overall, the clusters reflected distinct combinations of glycemic burden, insulin resistance, and β-cell functional capacity, underscoring the metabolic heterogeneity at the time of diagnosis.

To complement the standardized radar profiles, **Table 3** summarizes the clinical and metabolic characteristics of each cluster using the original variable values. Significant differences were observed across all measured parameters, including BMI, HbA1c, basal C-peptide, HOMA2-%B and HOMA2-IR (all p < 0.001). The tabulated data confirmed the distinct metabolic phenotypes suggested by the radar plots. Cluster 1 represented a relatively balanced metabolic profile with moderate glycemic impairment and preserved insulin sensitivity. Cluster 2 was characterized by the highest basal C-peptide concentrations together with marked insulin resistance. Cluster 3 exhibited the most severe hyperglycemia accompanied by profound β-cell dysfunction. In contrast, Cluster 4 was distinguished by the highest BMI, marked insulin resistance, relatively preserved β-cell function, and comparatively lower HbA1c levels.

**Table 3:** Clinical characteristics of the four clusters.

Parameter	Cluster 1 (n=75)	Cluster 2 (n=23)	Cluster 3 (n=45)	Cluster 4 (n=35)	p-value
Male, n (%)	33 (44.0)	17 (73.9)	27 (60.0)	23 (65.7)	0.029
BMI, kg/m²	27.5 [25.1; 30.4]	30.8 [27.2; 37.2]	29.3 [25.8; 31.8]	35.6 [31.8; 38.5]	<0.001
HbA1c, %	7.26 [6.50; 8.87]	8.75 [6.59;11.12]	11.61 [10.78;13.78]	6.51 [6.50; 6.78]	<0.001
Basal C-peptide,ng/mL	1.98 [1.39; 2.72]	5.53 [4.10; 6.37]	1.63 [1.02; 2.42]	3.13 [2.49; 4.37]	<0.001
HOMA2-%B	50.3 [33.4; 90.7]	78.8 [40.2;143.7]	20.5 [12.05;33.55]	127.5 [96.3;183.9]	<0.001
HOMA2-%S	68.3 [47.4;102.5]	27.4 [15.5; 55.9]	66.0 [41.0;108.5]	33.2 [27.4; 53.4]	<0.001
HOMA2-IR	1.46 [0.98; 2.11]	3.65 [2.20; 6.10]	1.52 [0.94; 2.59]	3.01 [1.81; 3.65]	<0.001

Data are presented as median [IQR]. Between-cluster differences were assessed using the Kruskal–Wallis test. Categorical variables were compared using Pearson’s χ².

Sex distribution differed significantly across the four metabolic clusters (χ² = 9.02, p = 0.029). Cluster 1 had the lowest proportion of men (44.0%), whereas Cluster 2 showed the highest proportion of men (73.9%). Men also predominated in Clusters 3 and 4 (60.0% and 65.7%, respectively), indicating that sex composition varied significantly across the identified metabolic phenotypes.

## 4 Discussion

This study aimed to characterize metabolic heterogeneity in newly diagnosed type 2 diabetes mellitus after exclusion of autoimmune diabetes and to determine whether clinically defined pathophysiological phenotypes correspond to naturally emerging metabolic clusters.

Our findings reinforce the concept that T2DM is not a single uniform disorder but a heterogeneous metabolic spectrum (13). Even at diagnosis, patients demonstrated divergent contributions of insulin resistance and insulin secretory dysfunction despite similar disease duration. This observation is consistent with the “palette model” of diabetes, which proposes that hyperglycemia arises from different combinations of pathophysiological pathways, including insulin resistance, impaired insulin secretion, adiposity-related mechanisms, and glucotoxicity (14). Sex distribution also differed across the identified metabolic clusters. This observation is biologically plausible and is consistent with established sex differences in adiposity, insulin sensitivity, and β-cell function, which are known to contribute to metabolic heterogeneity in type 2 diabetes (15).

More than half of the islet autoantibody-negative participants in our cohort (59%) exhibited HOMA2-IR values ≥1.6, indicating that insulin resistance represents the predominant pathophysiological mechanism in this population. In contrast, reduced basal C-peptide concentrations (<1.70 ng/mL), reflecting impaired β-cell secretory function, were observed in only 30.9% of patients, whereas preserved endogenous insulin secretion was present in the majority (69.1%). These findings suggest that the predominant metabolic phenotype of seronegative type 2 diabetes in our cohort is characterized by marked insulin resistance with relatively preserved β-cell function rather than primary β-cell failure. The HOMA2-IR threshold of 1.6 used in the present study is consistent with findings from the UKPDS 89 study, which included 4,344 individuals with newly diagnosed type 2 diabetes. In that cohort, the median HOMA2-IR was 1.6 [1.1;2.2] (16). Similarly, the basal C-peptide threshold of 1.70 ng/mL (0.57 nmol/L) was selected based on previously established clinical practice. This cutoff was initially introduced in Korean studies and was subsequently adopted for stratifying insulin secretory dysfunction in patients with T2DM across East Asian populations (5). These thresholds were derived from studies conducted in European and East Asian populations, as no population-specific validation studies are currently available for Central Asian populations. Future studies should determine whether population-specific cut-off values are warranted for this population.

When interpreted within the broader framework of data-driven diabetes stratification, our results extend the clustering paradigm introduced by Emma Ahlqvist et al. in the Swedish ANDIS cohort, which demonstrated that adult-onset diabetes can be divided into biologically meaningful subgroups with differential complication risks (17). Unlike the Swedish model, which incorporated autoantibody status and age at diagnosis as primary clustering variables, our analysis deliberately excluded autoimmune diabetes and focused exclusively on fasting-derived metabolic parameters: BMI, HbA1c, HOMA2-%B, HOMA2-IR, basal C-peptide. This approach allowed us to isolate metabolic heterogeneity within seronegative T2DM and minimize confounding by immune-mediated or age-driven separation. The absence of an autoimmune-dominant cluster in our cohort is therefore methodologically expected rather than conceptually contradictory. To our knowledge, this is the first study to apply an unsupervised clustering approach to newly diagnosed T2DM in the Kazakh population after systematic exclusion of autoimmune diabetes. Given known ethnic differences in insulin sensitivity and β-cell reserve, these findings contribute region-specific data to the global discussion on diabetes heterogeneity.

Recent international studies further demonstrate that data-driven subgroups of newly diagnosed type 2 diabetes reflect biologically meaningful heterogeneity rather than arbitrary statistical partitions. Replication studies in Chinese cohorts have confirmed the presence of MOD, MARD, SIDD, and SIRD phenotypes and demonstrated that these subtypes differ in cardiovascular genetic risk profiles and estimated Framingham risk, supporting their prognostic relevance (18–20). A recent cluster analysis in a Singaporean cohort of younger-onset type 2 diabetes further extended these findings, identifying distinct subgroups with differential pathophysiology and clinical outcomes, and thereby providing evidence directly relevant to Asian populations (21).

Moreover, secondary analyses of the MARCH randomized cohort revealed significant cluster-by-treatment interactions, with metformin preferentially lowering fasting glucose in MARD and acarbose exerting greater effects on postprandial glycemia in MOD, indicating subtype-specific therapeutic responses even within first-line oral agents (22). In South Asian populations, including North Indian cohorts, cluster distributions differ substantially from European populations, with a more prominent contribution of β-cell dysfunction accompanied by insulin resistance across clusters, underscoring ethnic variability in the relative weight of pathophysiological mechanisms (23). The concept of data-driven subclassification of type 2 diabetes has also been validated in several international cohorts, supporting the robustness of the ANDIS framework. For example, an analysis of over 20,000 patients with high cardiovascular risk from the DEVOTE, LEADER, and SUSTAIN-6 trials demonstrated that simplified clustering based on age, BMI, and HbA1c could reproduce ANDIS-like subgroups. In this analysis, the cluster resembling severe insulin-deficient diabetes showed the highest risk of cardiovascular mortality and diabetic nephropathy, confirming the prognostic relevance of metabolic stratification even in advanced stages of diabetes (24). These findings indicate that key pathophysiological phenotypes can be identified using a limited set of routinely available clinical variables. This feature may facilitate future translation of metabolic phenotyping into routine clinical settings without the need for specialized dynamic testing, imaging, or genetic profiling. Following external and prospective validation, the identified phenotypes could potentially form the basis for a simple clinical decision-support algorithm for stratifying newly diagnosed patients according to their predominant metabolic characteristics.

The four-cluster structure identified in our study supports the hypothesis that early T2DM can be decomposed along distinct metabolic axes. Similar to previous studies, we observed patterns corresponding to insulin resistance–predominant and β-cell dysfunction–predominant phenotypes, as well as intermediate or mixed metabolic profiles. These findings are consistent with pathophysiological models suggesting that insulin resistance and β-cell dysfunction evolve in parallel but at variable rates across individuals (25).

The moderate bootstrap stability observed for Clusters 2 and 3 suggests that the boundaries between these phenotypes are less distinct than those of Clusters 1 and 4. This may reflect the biological continuum between insulin resistance and β-cell dysfunction, whereby individuals with intermediate metabolic profiles may shift between adjacent phenotypes under resampling. Accordingly, Clusters 2 and 3 should be interpreted as metabolically informative but less sharply separated phenotypes, and their reproducibility requires confirmation in larger independent cohorts.

Potential clinical implications of the identified phenotypes. Interpreted within the framework of the above literature, the four phenotypes identified in our cohort may carry distinct, though hypothesis-generating, clinical implications. Cluster 1, with a balanced metabolic profile, may correspond to a subgroup in which standard first-line management is likely sufficient. Cluster 2, characterized by hyperinsulinemia and elevated BMI, phenotypically resembles the MOD/SIRD subgroup and may therefore share its reported preferential response to insulin-sensitizing and weight-directed therapies (22). Cluster 3, dominated by severe hyperglycemia and reduced β-cell function, approximates the SIDD-like profile associated in prior cohorts with the highest microvascular and cardiovascular risk, potentially justifying earlier consideration of β-cell-protective strategies (17, 24). The development of diabetes-related complications is likely to reflect mechanisms beyond metabolic dysfunction alone, including inflammatory processes; recent findings by Bulu et al., for example, demonstrated associations of Maresin 1 and CHI3L1 with renal dysfunction in diabetic nephropathy (26). Cluster 4, combining high BMI with preserved β-cell function and lower glycemic burden, may represent an early, potentially reversible metabolic stage in which intensive weight-directed intervention could plausibly modify disease trajectory. Prospective longitudinal validation is required before any of these implications can inform clinical decisions.

Taken together, these findings support the concept that type 2 diabetes represents a heterogeneous metabolic spectrum and highlight the importance of population-specific analyses for identifying dominant pathophysiological mechanisms across different ethnic groups.

## 5 Limitations

Several limitations should be acknowledged. First, the cross-sectional design precludes causal inference and does not permit assessment of longitudinal changes in metabolic phenotypes or their clinical consequences. Second, clustering was based on fasting-derived surrogate indices, which may not fully capture dynamic β-cell function or insulin secretory responses. Third, although the sample size was sufficient for internal validation, external validation in larger, independent, multicenter cohorts from Central Asian populations is required to confirm the reproducibility and generalizability of the identified metabolic phenotypes. Finally, because of the cross-sectional nature of the study, the present findings cannot establish whether these metabolic phenotypes differ with respect to the risk of microvascular or macrovascular complications, therapeutic responses, or long-term clinical outcomes. These potential clinical implications should therefore be regarded as hypotheses that require confirmation in future prospective longitudinal studies. Additionally, bootstrap-based internal validation demonstrated only moderate stability for Clusters 2 and 3 (mean Jaccard coefficients 0.715 and 0.650, respectively); therefore, the boundary between the insulin-resistant/hyperinsulinemic and the β-cell-deficient/hyperglycemic phenotypes should be interpreted with particular caution and confirmed in independent cohorts. Furthermore, the threshold values used to define insulin resistance (HOMA2-IR ≥1.6) and reduced β-cell secretory capacity (basal C-peptide <1.70 ng/mL) were adopted from European and East Asian cohorts, respectively, and have not, to our knowledge, been specifically validated in the Central Asian population; population-specific cut-off values remain to be established.

## Data Availability

The raw data supporting the conclusions of this article will be made available by the authors, without undue reservation.
