# Supplementary material for: Metabolic heterogeneity at the onset of type 2 diabetes: phenotypes of insulin resistance and β-cell function in the Kazakh population
Source: Front Endocrinol (Lausanne). 2026 Aug 25;17:1824856. doi: 10.3389/fendo.2026.1824856 (PMC13549826; doi:10.3389/fendo.2026.1824856)
Supplement: Supplementary file 1 [file Table1.docx]

## Table S1. Characteristics according to HOMA2-IR threshold

| Characteristic | HOMA2-IR <1.6  N = 73^1^ | HOMA2-IR ≥1.6  N = 105^1^ | p-value^2^ |
| --- | --- | --- | --- |
| Age | 52.00 [44.00; 61.00] | 51.00 [39.00; 59.00] | 0.4 |
| Sex |  |  | >0.9 |
| Male | 41 (56%) | 59 (56%) |  |
| Female | 32 (44%) | 46 (44%) |  |
| BMI | 28.10 [25.20; 32.30] | 30.50 [26.80; 34.70] | 0.006 |
| HbA1c | 7.95 [6.50; 10.79] | 7.73 [6.53; 10.53] | 0.7 |
|  | C-peptide ≥1.70 ng/mL  N = 123^1^ | C-peptide <1.70 ng/mL  N = 55^1^ | p-value^2^ |
| Age | 52.00 [42.00; 60.00] | 50.00 [41.00; 57.00] | 0.5 |
| Sex |  |  | 0.9 |
| Male | 70 (57%) | 30 (55%) |  |
| Female | 53 (43%) | 25 (45%) |  |
| BMI | 30.50 [26.80; 34.30] | 27.70 [24.60; 30.50] | <0.001 |
| HbA1c | 7.23 [6.50; 9.44] | 9.19 [6.70; 11.27] | 0.008 |
